# When low-order expansions fail and all higher-order contributions matter—basic example of the mean squared displacement for Brownian motion

**DOI:** 10.1140/epje/s10189-022-00232-z

**Published:** 2022-09-19

**Authors:** Andreas M. Menzel

**Affiliations:** Institut für Physik, Otto-von-Guericke-Universität Magdeburg, Universitätsplatz 2, 39106 Magdeburg, Germany

## Abstract

Hardly any theoretically formulated realistic problem can be solved exactly. Therefore, as a standard, we resort to approximations. In this context, expansions play a major role. We are used to relying on lowest-order expansions and confining our point of view accordingly. However, one should always bear in mind that such considerations may fail at some point. Here, we address a very common example situation, namely, the motion of a Brownian particle. We know that the associated mean-squared displacement in the long term increases linearly in time. Yet, when we take the Fokker–Planck approach in combination with a low-order expansion, the direct route towards this result fails. That is, in the expansion the term linear in time vanishes. Instead, the treatment requires consideration of *all* higher-order contributions. Together, they restore the linear increase in time. In this way, we stress that care is always mandatory when resorting to low-order expansions, and we present in a traceable way a route to solving the considered problem.

## Introduction

Claiming that in theoretical physics expansions in terms of power series are common almost sounds like an understatement. The harmonic oscillator, interpreted as the result of a lowest-order expansion in potential energy, is of textbook caliber, both in classical and quantum mechanics [1–3]. Moreover, we may regard Newtonian mechanics in general as an expansion of the special theory of relativity to low orders in velocity [3]. The same applies to hydrodynamics [4] in the context of the general theory of relativity [5] to low orders in velocity and mass density. Besides, the central equation of hydrodynamics, the Navier-Stokes equation, employs a lowest-order expansion in the symmetrized velocity gradient for the terms associated with viscous stress [4]. Textbook electrodynamics mostly is restricted to terms linear in the electric and magnetic fields [6]. Several famous treatments of elasticity theory put a major emphasis on quadratic terms in the energy density [7]. In many of these situations, if nonlinearities are considered, they are added as higher-order terms in the notion of a power series. Other examples concern generalized hydrodynamics [8, 9] or expansions in actually not small quantities such as space dimension [10]. All these approaches have proven highly successful and have their justification.

Despite this triumph one should always keep in mind that power series expansions are only an approximation and, depending on the context, may fail. In our minimal example, we refer to the stochastic motion of a Brownian particle when subject to linear friction and a stochastic driving force. We address this situation in the framework of the Fokker–Planck equation [11–14].

On short time scales, motion, if not completely overdamped, is ballistic. Yet, it becomes diffusive on longer scales. That is, the mean squared displacement increases linearly in time. We calculate the mean squared displacement using a power series. There, the term linear in time vanishes, which appears as a contradiction to the linear increase. As it turns out, to solve this riddle, all higher-order contributions of the power series need to be taken into account as well. Together, they restore the linear increase in time in the long-term limit.

The remaining contents are as follows. In Sect. 2, we recall the underlying stochastic Langevin and, after rescaling, associated Fokker–Planck equations. We show in Sect. 3 that a lowest-order expansion in time fails to predict the linear, diffusive time dependence of the mean squared displacement. Subsequently, we demonstrate in Sect. 4 how the addition of all higher-order contributions restores this linear dependence. A simple comparison with numerical integration of the Langevin equations and the resulting expression of the diffusion coefficient confirm our solution, see Sect. 5. We conclude in Sect. 6.

## Stochastic equations of motion

To keep our example as simple as possible, we address the stochastic motion of a single Brownian particle in one spatial dimension. There, we mark by *x* the space coordinate and by *v* the velocity. This leads us to the conventional Langevin-type equations of motion [15]

1 $$\begin{aligned} m\,\frac{\mathrm {d}v}{\mathrm {d}t}= & {} -\zeta \,v +\Gamma (t), \end{aligned}$$

2 $$\begin{aligned} \frac{\mathrm {d}x}{\mathrm {d}t}= & {} v, \end{aligned}$$

where *t* denotes time. Moreover, *m* is the mass of the Brownian particle and $\zeta$ represents the coefficient of linear friction that always acts against the current direction of motion. $\Gamma (t)$ corresponds to the stochastic force that the particle is exposed to at time *t*. As is common, we consider $\Gamma (t)$ to be distributed according to a Gaussian form and $\delta$-correlated in time, so that $\langle \Gamma (t)\rangle =0$ and $\langle \Gamma (t)\Gamma (t')\rangle =2K\,\delta (t-t')$. Thus, *K* sets the strength of the stochastic force acting on the Brownian particle. Physically, thermal fluctuations provide the background of this stochastic force. Therefore, *K* is set by the fluctuation–dissipation theorem, that is, $K=\zeta \,k_B T$, where $k_B$ is the Boltzmann constant and *T* denotes temperature.

Through well-known procedures, Eqs. (1) and (2) are transformed to the associated Fokker–Planck equation [13–15]. After rescaling, we obtain in dimensionless form

3 $$\begin{aligned} \partial _t f = \left\{ \! -v\,\partial _x + \partial _v v + \partial _v^2\, \right\} f. \end{aligned}$$

Here, $f=f(x,v,t)$ represents the probability distribution to find the particle at time *t* with velocity *v* at position *x*. Normalization implies $\int _{-\infty }^{\infty }\mathrm {d}x\int _{-\infty }^{\infty }\mathrm {d}v \,f(x,v,t)=1$. We have rescaled *x* by $(Km/\zeta ^3)^{1/2}$, *v* by $(K/m\zeta )^{1/2}$, *t* by $m/\zeta$, and *f* by $\zeta ^2/K$, where the latter is necessary to maintain the normalization condition. Overall, there is no free parameter left in Eq. (3) so that our subsequent considerations apply to any Brownian particle.

Without loss of generality, we consider the Brownian particle to be located at time $t=0$ at position $x=0$. In this way, we specify as an initial condition for the probability distribution

4 $$\begin{aligned} f_0:=f(x,v,t=0)=\frac{1}{\sqrt{2\pi }}\mathrm {e}^{-\frac{1}{2}v^2}\delta (x). \end{aligned}$$

Again, $\delta$ denotes the Dirac delta function. The exponential represents the equilibrium Boltzmann velocity distribution in our rescaled units.

## Lowest order in time

We know that in the long term the mean squared displacement of a Brownian particle increases linearly in time. Thus, it is a tempting idea to expand the corresponding expression to lowest order in time and in this way determine the associated diffusion coefficient according to the relation $\langle x^2\rangle =2Dt$.

Along these lines, we proceed via the formal solution of Eq. (3),

5 $$\begin{aligned} f=\mathrm {e}^{t\left( -v\,\partial _x + \partial _v v + \partial _v^2\right) }f_0. \end{aligned}$$

Thus, we find for the mean squared displacement

6 $$\begin{aligned} \langle x^2\rangle= & {} \int _{-\infty }^{\infty }\mathrm {d}x \int _{-\infty }^{\infty }\mathrm {d}v \, x^2\, \mathrm {e}^{t\left( -v\,\partial _x + \partial _v v + \partial _v^2\right) }f_0 \nonumber \\= & {} \int _{-\infty }^{\infty }\mathrm {d}x \int _{-\infty }^{\infty }\mathrm {d}v \, f_0\, \mathrm {e}^{t\left( v\,\partial _x - v\partial _v + \partial _v^2\right) }x^2, \end{aligned}$$

where we used partial integration to shift the exponential operator from $f_0$ to $x^2$ (see the “Appendix” for details).

In order to identify the lowest order in time *t*, we expand the exponential. To zeroth order in *t*, inserting Eq. (4), we obtain on the second line of Eq. (6)

7 $$\begin{aligned} \int _{-\infty }^{\infty }\mathrm {d}x \int _{-\infty }^{\infty }\mathrm {d}v \, f_0\,x^2 = 0, \end{aligned}$$

which results from the Dirac delta function in $f_0$. Next, to first order in *t*, we might expect a nonvanishing contribution. After all, we know that the mean squared displacement in the end increases linearly in time. However, we find

8 $$\begin{aligned}&{ \int _{-\infty }^{\infty }{\mathrm {d}}x \int _{-\infty }^{\infty }{\mathrm {d}}v \, f_0\, {t\left( v\partial _x - v\partial _v + \partial _v^2\right) }x^2} \nonumber \\&\quad = \int _{-\infty }^{\infty }{\mathrm {d}}x \int _{-\infty }^{\infty }{\mathrm {d}}v \, f_0\,t\,2xv \nonumber \\&\quad = 0 \end{aligned}$$

which follows again by inserting Eq. (4). The mean squared displacement therefore does *not* increase linearly in time to lowest order. Instead, we find to second order in time *t* together with Eq. (4)

9 $$\begin{aligned}&{ \int _{-\infty }^{\infty }{\mathrm {d}}x \int _{-\infty }^{\infty }{\mathrm {d}}v \, f_0\, \frac{1}{2}{t^2\left( v\partial _x - v\partial _v + \partial _v^2\right) }^2x^2,} \nonumber \\&\quad =\frac{1}{2}t^2\int _{-\infty }^{\infty }{\mathrm {d}}x \int _{-\infty }^{\infty }{\mathrm {d}}v \, f_0\, \left( 2v^2-2xv\right) \nonumber \\&\quad =t^2. \end{aligned}$$

Thus, the lowest nonvanishing order of the mean squared displacement is *quadratic* in time, not linear. On the one hand, this is reasonable because it identifies the initial ballistic regime of motion for the Brownian particle, which later crosses over to diffusive motion. On the other hand, this triggers the question of how, then, at later times, can the motion become diffusive, that is, linear in time, if the linear term obviously vanishes?

## Higher-order contributions

It turns out that, in order to answer this question, we need to calculate *all* higher-order contributions. To this end, we consider the power series expansion of the exponential

10 $$\begin{aligned} {\mathrm {e}}^{t\left( v\partial _x-v\partial _v+\partial _v^2\right) }x^2 = \sum _{n=0}^{\infty } \frac{1}{n!}t^n \left( v\partial _x\!-\!v\partial _v\!+\!\partial _v^2\right) ^nx^2.\nonumber \\ \end{aligned}$$

Applying the operator in brackets once to $x^2$, we find for the *n*th term

11 $$\begin{aligned} 2\frac{1}{n!}t^n\left( v\partial _x-v\partial _v+\partial _v^2\right) ^{n-1}xv; \end{aligned}$$

twice, we find

12 $$\begin{aligned} 2\frac{1}{n!}t^n\left( v\partial _x-v\partial _v+\partial _v^2\right) ^{n-2} \left( v^2-xv\right) ; \end{aligned}$$

three times, we find

13 $$\begin{aligned} 2\frac{1}{n!}t^n\left( v\partial _x-v\partial _v+\partial _v^2\right) ^{n-3} \left( -3v^2+xv+2\right) ;\nonumber \\ \end{aligned}$$

four times, we find

14 $$\begin{aligned} 2\frac{1}{n!}t^n\left( v\partial _x-v\partial _v+\partial _v^2\right) ^{n-4} \left( 7v^2-xv-6\right) ; \end{aligned}$$

five times, we find

15 $$\begin{aligned} 2\frac{1}{n!}t^n\left( v\partial _x-v\partial _v+\partial _v^2\right) ^{n-5} \left( -15v^2+xv+14\right) ;\nonumber \\ \end{aligned}$$

six times, we find

16 $$\begin{aligned} 2\frac{1}{n!}t^n\left( v\partial _x-v\partial _v+\partial _v^2\right) ^{n-6} \left( 31v^2-xv-30\right) ;\nonumber \\ \end{aligned}$$

seven times, we find

17 $$\begin{aligned} 2\frac{1}{n!}t^n\left( v\partial _x-v\partial _v+\partial _v^2\right) ^{n-7} \left( -63v^2+xv+62\right) ;\nonumber \\ \end{aligned}$$

and so on. Thus, Eqs. (11)–(17) directly provide the results for the terms for $n=1$–7 by inserting these numbers, respectively.

Our claim from inspecting Eqs. (11)–(17) is that the *n*th term in the power series of Eq. (10) for $n\ge 2$ is given by

18 $$\begin{aligned}&{2\frac{1}{n!}t^n\Big [ (-1)^{n-1}xv +(-1)^n\left( 2^{n-1}-1\right) v^2} \nonumber \\&\quad +(-1)^{n-1}\left( 2^{n-1}-2\right) \Big ]. \end{aligned}$$

We prove this claim by induction in that we apply once more the operator $\left( v\partial _x-v\partial _v+\partial _v^2\right)$ to the term in square brackets in Eq. (18),

19 $$\begin{aligned}&{\left( v\partial _x-v\partial _v+\partial _v^2\right) \Big [ (-1)^{n-1}xv }+(-1)^n\left( 2^{n-1}-1\right) v^2\nonumber \\&\qquad +(-1)^{n-1}\left( 2^{n-1}-2\right) \Big ] \nonumber \\&\quad = (-1)^{n-1}v^2 +(-1)^n xv -2(-1)^n\left( 2^{n-1}-1\right) v^2 \nonumber \\&\qquad +2(-1)^n\left( 2^{n-1}-1\right) \nonumber \\&\quad =(-1)^{(n+1)-1}xv +(-1)^{n+1}\left( 2^{(n+1)-1}-1\right) v^2 \nonumber \\&\qquad +(-1)^{(n+1)-1}\left( 2^{(n+1)-1}-2\right) . \end{aligned}$$

This result equals the term in square brackets in Eq. (18) with *n* increased by 1, which completes the induction. Moreover, Eq. (18) for $n=2$ leads to the same result as Eq. (12) for $n=2$. Together, we have proven that Eq. (18) provides the correct result for terms $n\ge 2$ in the power series of Eq. (10).

Summarizing, we find

20 $$\begin{aligned}&{ \mathrm {e}^{t\left( v\partial _x-v\partial _v+\partial _v^2\right) }x^2}\nonumber \\&\quad = x^2 + 2txv + 2\sum _{n=2}^{\infty }\frac{1}{n!}t^n \Big [ (-1)^{n-1}xv \nonumber \\&\qquad +(-1)^n\left( 2^{n-1}-1\right) v^2 \nonumber \\&\qquad +(-1)^{n-1}\left( 2^{n-1}-2\right) \Big ]. \end{aligned}$$

This expression is inserted into Eq. (6). Together with $f_0$, we integrate it over *x* and *v*. Due to the form of $f_0$, see Eq. (4), the integration of *x* and $x^2$ yields 0. The integration of $v^2$ together with $f_0$ yields 1.

Combining Eqs. (4), (6), and (20), we therefore obtain

21 $$\begin{aligned} \langle x^2\rangle= & {} 2\sum _{n=2}^{\infty }\frac{1}{n!}t^n(-1)^n \Big [ 2^{n-1}-1-(2^{n-1}-2) \Big ] \nonumber \\= & {} 2\sum _{n=2}^{\infty }\frac{1}{n!}(-t)^n. \end{aligned}$$

In this expression, we identify the power series expansion of an exponential, *except that* the zeroth- and first-order contributions are missing. The latter observation is crucial. It takes us back to the result in Sect. 3 of vanishing linear order in time of the mean squared displacement. Now, however, we can resolve this issue.

Formally, we may provide the zeroth- and first-order contributions by adding them, if we directly subtract them again. In this way, we can reformulate the sum to an exponential form,

22 $$\begin{aligned} \langle x^2\rangle= & {} -2 +2t +2\sum _{n=0}^{\infty }\frac{1}{n!}(-t)^n \nonumber \\= & {} 2\left( t -1 +\mathrm {e}^{-t}\right) . \end{aligned}$$

Thus, introducing the exponential adds the term linear in time to the mean squared displacement.

This is our central result. It now *does* contain explicitly the linear dependence of the mean squared displacement on time, which identifies diffusive behavior. This is true particularly at elevated times when the exponential dependence has decayed towards zero. At short times, we again find the ballistic behavior $\langle x^2\rangle \approx t^2$ to lowest order, as already identified in Sect. 3.

Interpreting Eq. (22) and the way that guided us towards it leads us to the following observation. The linear dependence on time of the mean squared displacement is *not* found as a low-order term from its power series expansion. Instead, in the power series expansion, the term linear in time initially *vanishes*, which may come as a bit of a surprise if one is used to working with power series expansions on a daily basis. Remarkably, however, *all higher-order* terms act together to *restore* the linear dependence. We have revealed this context in Eq. (22) by completing the power series expansion of the exponential function. Therefore, we note that all higher-order terms here are necessary and contribute to obtain the low-order linear dependence on time of the mean squared displacement.

## Confirmation of our result

To briefly test our result in Eq. (22), we perform straightforward particle-based simulations of Eqs. (1) and (2), after rescaling them to dimensionless units. The values of the stochastic force are drawn from a random number generator [16] and cast to a Gaussian distribution [17]. We calculate the trajectories using simple Euler forward integration in time and a time step of $\Delta t=0.001$. To obtain the mean squared displacement, we average over $10^6$ particle trajectories.

**Fig. 1:**
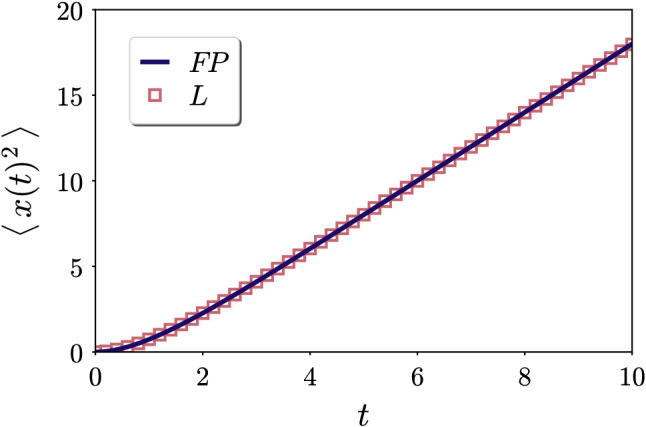
Mean squared displacement for the motion of a Brownian particle. We compare the functional form obtained in Eq. (22) by analytical calculation from the Fokker–Planck equation (“*FP*”, solid line) involving all higher-order contributions with results from particle-based simulations of the Langevin representation (“*L*”, data points) in Eqs. (1) and (2). Both types of approach lead to identical curves

Our results from the particle-based simulations are shown in Fig. 1 together with our functional form displayed in Eq. (22). Both approaches lead to identical curves, which verifies our calculation and interpretation.

A final check concerns the diffusion coefficient *D*. Via the definition $\langle x^2\rangle =2Dt$, we obtain from the term linear in time *t* in Eq. (22) the value $D=1$ in rescaled units. Scaling the equation $\langle x^2\rangle =2Dt$ back to dimensionful spatial positions *x* and times *t*, we find

23 $$\begin{aligned} D=\frac{k_BT}{\zeta }. \end{aligned}$$

This is the correct result according to the fluctuation–dissipation relation [15].

## Conclusions

The main purpose of our work is to demonstrate on a very basic, illustrative example that expansions to given finite orders can lead to misleading results. Specifically, we address the mean squared displacement of an individual particle performing Brownian motion. We calculate this quantity in an analytical, yet unconventional way. To this end, the formal solution to the associated Fokker–Planck equation for the probability distribution in exponential form is employed.

Expanding this solution demonstrates that the low-order contribution linear in time vanishes. This observation contradicts our experience that the mean squared displacement for the diffusive behavior of Brownian particles does grow linearly in time. Remarkably, as we have demonstrated, we instead need to evaluate *all* higher-order contributions. Together, they to lowest order restore the linear time dependence characteristic for the diffusive behavior.

We briefly recapitulate the physical reason for the failure of reproducing the linear growth of the mean squared displacement in time by the low-order expansion. In our situation, we correctly identified the initial but transient ballistic behavior of the Brownian particle. It is this behavior at the earliest times that is associated with the lowest-order temporal terms, correctly identified on the smallest time scales of the problem. Thus, caution is generally appropriate when dealing with series expansions in situations or systems featuring transient initial or intermediate regimes. For clarity, we repeat that, in our investigation, we did not assume the dynamics of the Brownian particle to be completely overdamped. Instead, we kept the inertial contribution.

Our motivation behind this presentation is twofold. On the one hand, we wish to outline the unconventional way of calculating the mean squared displacement. Although a bit more complex, this route may open the possibility to calculate diffusion coefficients also in the context of stochastic motion under nonlinear friction [18–25]. On the other hand, we wish to stress, using this basic example, that we always need to scrutinize the strategies of solution that we apply. Depending on the context, this statement even applies to well-established and straightforward basic expansion techniques. In the present framework, we demonstrate how the initial question of vanishing linear order can be solved by including all higher-order contributions.

## Data Availability

This manuscript has associated data in a data repository. [Author’s comment: All data generated and interpreted during this study are included in this published article, see the equations in the text and the data points in Fig. 1.]

## Appendix

In this appendix, we briefly illustrate an explicit way to perform the partial integration in Eq. (6). We first expand the exponential as

24 $$\begin{aligned}&{ \int _{-\infty }^{\infty }\mathrm {d}x \int _{-\infty }^{\infty }\mathrm {d}v \, x^2\, \mathrm {e}^{t\left( -v\,\partial _x + \partial _v v + \partial _v^2\right) }f_0}\nonumber \\&\quad = \int _{-\infty }^{\infty }\mathrm {d}x \int _{-\infty }^{\infty }\mathrm {d}v \, x^2\nonumber \\&\qquad \times \sum _{n=0}^{\infty }\frac{1}{n!}\,t^n \left( -v\partial _x+\partial _vv+\partial _v^2\right) ^n f_0. \end{aligned}$$

In each order *n* ($n\ge 1$), we start from the left-most operator $\left( -v\partial _x+\partial _v v+\partial _v^2\right)$ and shift it by partial integration to $x^2$ under the two integrals, where we keep all $\partial _v$-derivatives (even if they vanish),

25 $$\begin{aligned}&{ \int _{-\infty }^{\infty }\mathrm {d}x \int _{-\infty }^{\infty }\mathrm {d}v \, x^2 \frac{1}{n!}\,t^n\left( -v\partial _x+\partial _v v+\partial _v^2\right) ^n f_0}\nonumber \\&\quad = \int _{-\infty }^{\infty }\mathrm {d}x \int _{-\infty }^{\infty }\mathrm {d}v \, x^2 \frac{1}{n!}\,t^n\left( -v\partial _x+\partial _v v+\partial _v^2\right) \nonumber \\&\qquad \times \left( -v\partial _x+\partial _v v+\partial _v^2\right) ^{n-1} f_0 \nonumber \\&\quad = \int _{-\infty }^{\infty }\mathrm {d}x \int _{-\infty }^{\infty }\mathrm {d}v \, \frac{1}{n!}\,t^n \bigg [ \left( v\partial _x-v\partial _v +\partial _v^2\right) x^2 \bigg ]\nonumber \\&\qquad \times \left( -v\partial _x+\partial _v v+\partial _v^2\right) ^{n-1} f_0. \end{aligned}$$

$f_0$ and its derivatives vanish at infinity, both in *x*- and *v*-direction, thus no contributions arise from there. We continue in the same way successively from the left to the right with the remaining $n-1$ operators $\left( -v\partial _x+\partial _v v+\partial _v^2\right) ^{n-1}$ on the last line of Eq. (25). Each operator $\left( -v\partial _x+\partial _v v+\partial _v^2\right)$ becomes $\left( v\partial _x-v\partial _v +\partial _v^2\right)$ upon partial integration. In this way, we finally obtain from Eq. (24) via Eq. (25)

26 $$\begin{aligned}&{ \int _{-\infty }^{\infty }\mathrm {d}x \int _{-\infty }^{\infty }\mathrm {d}v \, f_0\, \sum _{n=0}^{\infty } \frac{1}{n!}\,t^n \left( v\partial _x-v\partial _v +\partial _v^2\right) ^n x^2 } \nonumber \\&\quad = \int _{-\infty }^{\infty }\mathrm {d}x \int _{-\infty }^{\infty }\mathrm {d}v \, f_0\, \mathrm {e}^{t\left( v\partial _x-v\partial _v +\partial _v^2\right) }x^2. \end{aligned}$$

## References

[1] Fetter AL, Walecka JD (2003). Theoretical Mechanics of Particles and Continua.

[2] Landau LD, Lifshitz EM (2003). Quantum Mechanics.

[3] Goldstein H, Safko C, Poole S (2014). Classical Mechanics.

[4] Landau LD, Lifshitz EM (2013). Fluid Mechanics.

[5] Weinberg S (1972). Gravitation and Cosmology: Principles and Applications of the General Theory of Relativity.

[6] Jackson JD (1999). Classical Electrodynamics.

[7] Landau LD, Lifshitz EM (1986). Theory of Elasticity.

[8] Martin PC, Parodi O, Pershan PS (1972). Unified hydrodynamic theory for crystals, liquid crystals, and normal fluids. Phys. Rev. A.

[9] H. Pleiner, H.R. Brand, in *Pattern formation in liquid crystals*. ed. by A. Buka, L. Kramer (Springer, New York, 1996), pp. 15–67

[10] Goldenfeld N (1992). Lectures on Phase Transitions and the Renormalization Group.

[11] Fokker AD (1914). Die mittlere Energie rotierender elektrischer Dipole im Strahlungsfeld. Ann. Phys..

[12] M. Planck, *Sitzungsberichte der Königlich Preussischen Akademie der Wissenschaften*, **7**, 324–341 (1940)

[13] Risken H (1996). The Fokker-Planck Equation: Methods of Solution and Applications.

[14] Zwanzig R (2001). Nonequilibrium Statistical Mechanics.

[15] Kubo R, Toda M, Hashitsume N (1991). Statistical Physics II: Nonequilibrium Statistical Mechanics.

[16] Matsumoto M, Nishimura T (1998). Mersenne twister: a 623-dimensionally equidistributed uniform pseudo-random number generator. ACM Trans. Model. Comput. Sim. (TOMACS).

[17] Flannery BP, Press WH, Teukolsky SA, Vetterling W (1992). Numerical Recipes in C.

[18] de Gennes PG (2005). Brownian motion with dry friction. J. Stat. Phys..

[19] Hayakawa H (2005). Langevin equation with Coulomb friction. Physica D: Nonlinear Phenom..

[20] Menzel AM, Goldenfeld N (2011). Effect of Coulombic friction on spatial displacement statistics. Phys. Rev. E.

[21] Touchette H, Van der Straeten E, Just W (2010). Brownian motion with dry friction: Fokker–Planck approach. J. Phys. A: Math. Theor..

[22] P.S. Goohpattader, M.K. Chaudhury, Diffusive motion with nonlinear friction: apparently Brownian. J. Chem. Phys. **133**, 024702 (2010) 10.1063/1.3460530 20632765

[23] A.M. Menzel, Velocity and displacement statistics in a stochastic model of nonlinear friction showing bounded particle speed. Phys. Rev. E **92**, 052302 (2015) 10.1103/PhysRevE.92.052302 26651690

[24] Das P, Puri S, Schwartz M (2017). Single particle Brownian motion with solid friction. Eur. Phys. J. E.

[25] Menzel AM (2022). Statistics for an object actively driven by spontaneous symmetry breaking into reversible directions. J. Chem. Phys..

